# Tuning Vibrational Coupling Strength Between Hyperbolic Media and Organic Molecules

**DOI:** 10.1002/nap2.70051

**Published:** 2026-03-24

**Authors:** Courtney L. Ragle, John E. Buchner, In‐Ho Lee, Youngji Kim, Byung‐Il Noh, Saurabh Dixit, Joseph Matson, Jiahan Li, Siyuan Dai, J. H. Edgar, Sang‐Hyun Oh, Lauren E. Buchanan, Joshua D. Caldwell

**Affiliations:** ^1^ Interdisciplinary Materials Science Program Vanderbilt University Nashville Tennessee USA; ^2^ Department of Mechanical Engineering Vanderbilt University Nashville Tennessee USA; ^3^ Department of Chemistry Vanderbilt University Nashville Tennessee USA; ^4^ Center for Quantum Technology Korea Institute of Science and Technology (KIST) Seoul South Korea; ^5^ Department of Mechanical Engineering Auburn University Auburn Alabama USA; ^6^ Tim Taylor Department of Chemical Engineering Kansas State University Manhattan Kansas USA; ^7^ Department of Electrical and Computer Engineering University of Minnesota Minneapolis Minnesota USA; ^8^ Sensorium Technological Laboratories Nashville Tennessee USA

**Keywords:** anti‐crossing, hybrid polariton, molecular polariton, natural hyperbolic material, passive tuning, phonon polariton, polariton dispersion, rabi splitting, s‐SNOM, vibrational strong coupling

## Abstract

In the field of molecular polaritonics, stronger coupling between vibrational modes and cavity systems is highly sought after. The coupling strength in these systems scales inversely with cavity mode volumes (V_m_), yet traditional Fabry–Perot cavities are diffraction‐limited. To overcome this limitation, we employ hyperbolic hexagonal boron nitride (hBN) to enable subdiffractional Fabry–Perot‐like modes. In this work, we use scattering‐type scanning near‐field optical microscopy (s‐SNOM) to directly image polariton propagation and quantify vibrational strong coupling to weak C‐H bending modes in poly(methyl methacrylate) (PMMA). The isotopic composition of the hBN layer dictates the frequency range of the material's Reststrahlen band, with the strongest coupling arising when the TO phonon is closest in energy to the molecular vibrational mode. We further characterize how coupling strengths scale with the thickness of the PMMA and hBN layers, as the PMMA layer thickness dictates the number of molecules available to fill an evanescent mode volume and the thickness of the hBN dictates the size of the evanescent mode volume. Ultimately, this study demonstrates that reduced mode volumes and a more complete understanding of the coupling mechanisms to tune and control the interactions are crucial to advancement in the field.

## Graphical abstract

Molecular polaritons, hybrid modes formed by vibrational bond modes and optical modes, typically in cavities, are investigated with the intent of inducing stronger coupling for the purpose of targeted control in a variety of application spaces. Here, we focus on decreasing mode volumes and assessing a variety of tuning parameters to increase coupling strength in systems consisting of subdiffractional materials paired with organic polymer.

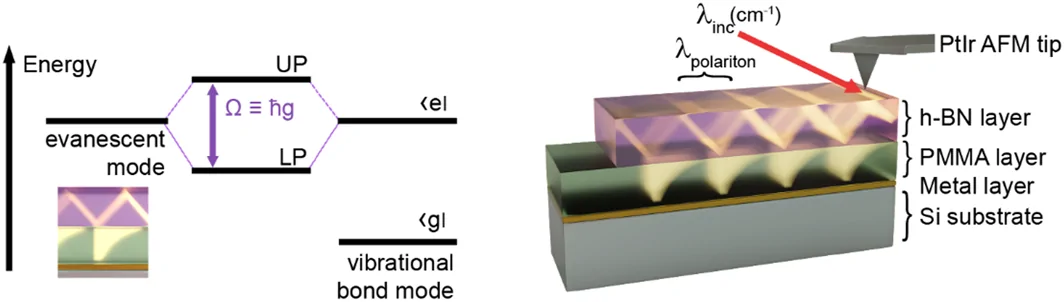

## 1 Introduction

Molecular polaritons, unique eigenstates formed by the hybridization of light and matter quantum states, have the potential to alter the fundamental physical and chemical properties of molecular systems. In particular, recent progress in the field of vibrational polaritonics has demonstrated altered vibrational lifetimes, chemical reaction rates, and chemical reaction pathways, laying the groundwork for light‐based control over chemical reaction mechanisms [1, 2, 3, 4, 5, 6, 7, 8, 9]. Such control will have significant impacts on both chemical research and industry by opening new synthetic pathways and enhancing the efficiency of existing ones, thus increasing yields and enabling the synthesis of previously unattainable molecules, while also promising cost reductions for large‐scale reactions. The coupling of molecular vibrations with an optical cavity (Figure 1A) is referred to as vibrational strong coupling (VSC) in systems where the energy exchange between the atomic oscillations and the optical cavity mode outlasts the energy dissipation (losses to the local environment) of the constituent modes [7, 8, 9, 10, 11, 12, 13, 14]. The coupling strength, ℏg(≡Ω) (Figure 1B), defined as the splitting between energy eigenstates, is characterized using the relationship:

(1) $$\hbar g\propto \mu \;\sqrt{\frac{N\hbar \omega }{\varepsilon V_{m}}}$$

where ω is the resonance frequency, N is the number of molecules in the cavity, ℏ is the reduced Planck's constant, μ is the molecular transition dipole strength, ε is the dielectric constant of the cavity material, and $V_{m}$ is the mode volume of the cavity [9, 10, 13, 14, 15].

**FIGURE 1:**
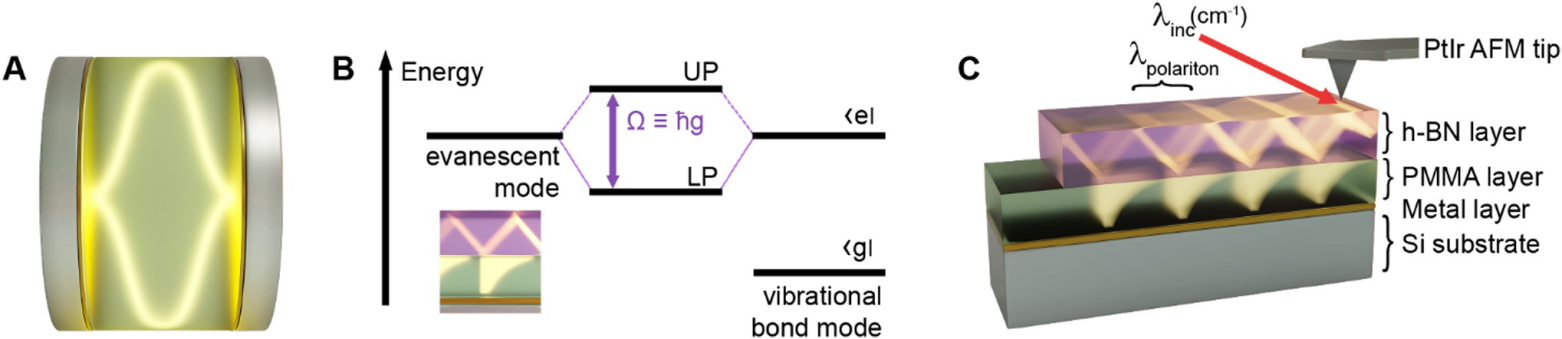
(A) Energy diagram depicting coupling between the propagated polariton evanescent mode and the targeted vibrational bond mode in PMMA. (B) Basic schematic of a Fabry–Perot cavity. (C) Schematic of the cavity design used, which includes a silicon substrate coated with a layer of gold, topped with a layer of PMMA of varied thicknesses, and a flake of hBN that was varied in both isotopic composition and thickness.

Traditionally, molecular polaritons have been studied using Fabry–Perot (FP) resonant cavities formed by two mirrors or two distributed Bragg reflectors (DBRs) with a finely tuned gap distance (Figure 1a). Although FP cavities can be employed to manipulate chemical reaction rates and even outcomes [2, 10, 11, 16, 17, 18], FP cavity lengths cannot be reduced below the diffraction limit (several μm in the infrared region), limiting the smallest $V_{m}$ to thousands of μm^3^. Thus, VSC in FP cavity systems is typically limited to molecules with large μ, such as metal carbonyls. Although these model cavity systems have provided significant and fundamental insights into VSC, novel approaches are needed to achieve stronger coupling with a more diverse array of organic and biological molecules to realize the full potential of polariton‐modified chemistry [1, 2, 8].

In this work, we move beyond traditional FP cavities, exploring a novel hybrid polaritonic system that enables subdiffractional FP‐like cavities. Such highly confined optical modes can be supported within hyperbolic materials, which are highly anisotropic materials where at least one principal axis exhibits a negative dielectric permittivity tensor component and another exhibits a positive dielectric permittivity tensor component. Hyperbolic modes enable the volume confinement of deeply subdiffractional light waves that propagate at fixed frequency‐dependent propagation angles, resulting in FP‐like behavior, with the wavelength of interest being that of the hyperbolic polariton [19]. Building on limited prior studies in which these types of systems are used, we systematically explore the role of various cavity design parameters to optimize VSC. The evanescent fields of the subdiffractional polaritonic waves formed in the hyperbolic hBN layer are coupled to the weak vibrational modes of the polymer layer (Figure 1C) [20]. Hyperbolic materials used in this manner have coupled the evanescent field of the hBN hyperbolic phonon polaritons (HPhPs) with an organic material, 4,4′‐bis(N‐carbazolyl)‐1,1′‐biphenyl (CBP), demonstrating the concept of strong coupling between hyperbolic media and organic vibrational modes and validating the ability to probe vibrational modes remotely [9, 21]. Given that this coupling is both theoretically and physically possible, we aim to expand the working principles governing VSC in subdiffractional geometries by shifting the spectral frequency of the Reststrahlen band of hBN using different boron isotopes (^10^B and ^11^B) while also exploring the role of hyperbolic (hBN) layer thickness, organic polymer thickness, and substrate coating considerations, in an effort to establish the guiding principles of amplifying and suppressing VSC within the subdiffractional limit.

Beyond the benefits afforded by increasing VSC with subdiffractional cavities, this approach also induces VSC within weaker oscillators (reduced μ, Equation 1). Hyperbolic materials such as hexagonal boron nitride (hBN) [19, 22, 23, 24, 25], calcite [24, 26], Al_2_O_3_ [24, 27], β‐Ga_2_O_3_ [23, 24, 27], MoO_3_ [24, 28], and others [19, 22, 23, 24, 25, 26, 28, 29, 30, 31] offer two key modifications to the more typical surface polaritonic behavior: (1) The polaritonic evanescent fields are confined and propagate through the volume of the material, and (2) the angle of propagation within the material becomes frequency‐dependent (Supporting Information S1: Figures S1–S2). This results in the aforementioned Fabry–Perot‐like response, but with the corresponding path length dictated by the subdiffractional HPhP wavelength (in contrast to the free‐space value) and the path length dictated by the frequency‐dependent propagation direction. We can designate the hyperbolic behavior as either Type I hyperbolicity, which is observed when the material exhibits a single dielectric tensor component that is negative, or Type II hyperbolicity, where there are two negative components [19, 22, 24, 32]. The generalized dielectric permittivity tensor associated with hyperbolic behavior in hBN is given by the following equation:

(2) $${\bar{\varepsilon }}_{hBN}=\left(\begin{array}{ccc} {\pm \;\varepsilon }_{∥} & 0 & 0\\ 0 & \pm \;\varepsilon_{∥} & 0\\ 0 & 0 & {\mp \varepsilon }_{⊥} \end{array}\right)$$

The first two individual permittivity tensor components on the diagonal are identical, as the material is isotropic in the x‐y plane [20, 24, 33, 34]. Propagation of an electromagnetic field (wavevector, *k*) through a material is dictated by the dispersion relation associated with the material:

(3) $$\frac{{k_{x}}^{2}}{\varepsilon_{1}}+\frac{{k_{y}}^{2}}{\varepsilon_{2}}+\frac{{k_{z}}^{2}}{\varepsilon_{3}}=\frac{\omega^{2}}{c^{2}}$$

Taking the hyperbolic dielectric permittivity tensor into account, the dispersion relation for hBN becomes as follows:

(4) $$\frac{\left({k_{x}}^{2}+{k_{y}}^{2}\right)}{\varepsilon_{∥}}+\frac{{k_{z}}^{2}}{\varepsilon_{⊥}}=\frac{\omega^{2}}{c^{2}}$$

where hBN offers an exemplary hyperbolic material in that it supports both Type I and Type II hyperbolicity in spectral regions separated by a large frequency gap, thereby allowing each region of hyperbolicity to be explored in separate application spaces and experiments [35].

As noted above, hyperbolicity results in volume‐confined polariton modes with frequency‐dependent ray‐like propagation angles in the material (Figure 1C). The propagation direction and the thickness dictate the path length for the hyperbolic modes; this gives rise to Fabry–Perot‐like cavity behavior in a deeply subdiffractional geometry, thereby substantially reducing $V_{m}$ while maintaining similar or stronger hybridization between the two modes. A key feature of hyperbolicity is the emergence of a spectral Reststrahlen band, which exhibits reflective behavior within a frequency range that is bounded by the lower‐frequency transverse optical (TO) and higher‐frequency longitudinal optical (LO) phonon modes, as shown in the dispersion plots in Figure 2B,C [19, 22, 25, 27, 34, 36]. Within this Reststrahlen band, the real part of the dielectric permittivity becomes negative, thereby enabling the material to support phonon polaritons. The evanescent field induced by this phonon polariton can then strongly couple to a molecular vibrational mode [9, 12, 22, 23, 37, 38, 39].

**FIGURE 2:**
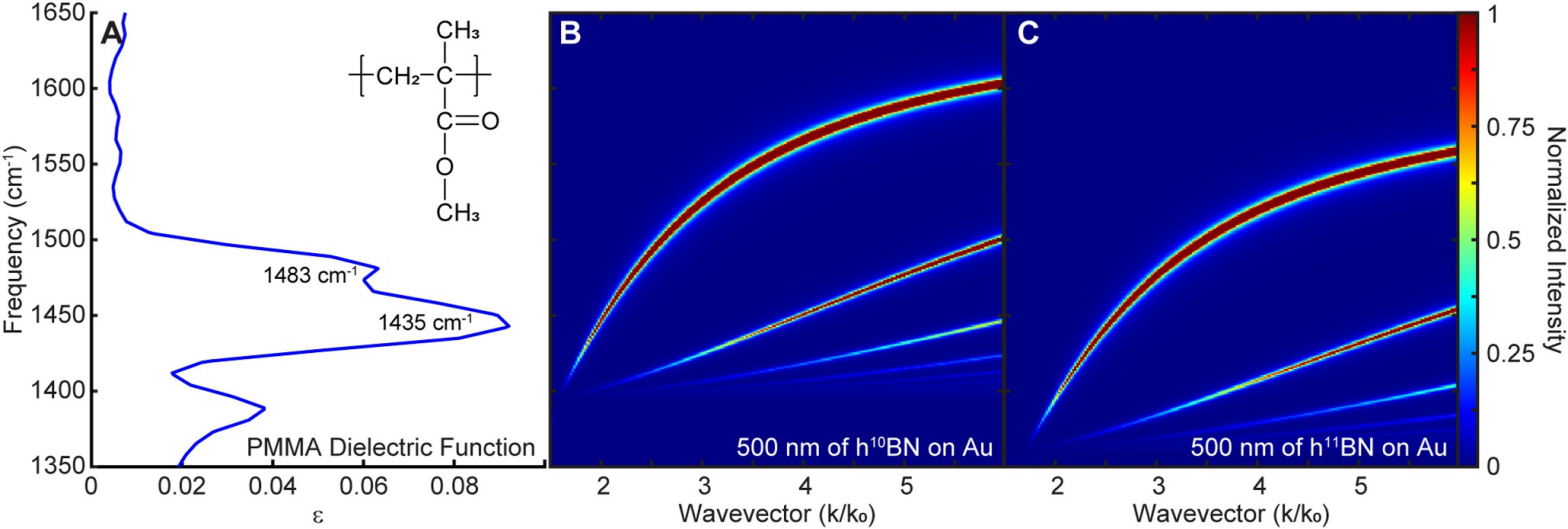
(A) Dielectric function of PMMA plotted over the frequency range encompassing the upper Reststrahlen bands of h^10^BN and h^11^BN, as seen in Panels (B) and (C), respectively, with an inset depicting the molecular structure of PMMA. (B) Polariton dispersion plot for a 500‐nm thick h^10^BN flake on gold with a TO phonon mode at 1394 cm^−1^ and an LO phonon mode at 1638 cm^−1^ encompassing the upper Reststrahlen band for the material. (C) Polariton dispersion plot for a 500‐nm thick h^11^BN flake on gold with a TO phonon mode at 1360 cm^−1^ and an LO phonon mode at 1599 cm^−1^ encompassing the upper Reststrahlen band for the material.

Although VSC to the strong carbonyl stretching mode of PMMA located spectrally at approximately 1730 cm^−1^ was achieved previously using FP cavities [40, 41], here we target the weaker bending modes of CH_2_ in the polymer backbone and CH_3_ in the side groups, which absorb at 1435 and 1483 cm^−1^, respectively [42]. These modes fall within the upper Reststrahlen bands of hBN, as well as the isotopes h^10^BN and h^11^BN (Figure 2), where Type II hyperbolicity is exhibited. Our cavity design (Figure 1C) allows the thickness of both the polymer layer and the hBN flake to be varied to provide experimental control over the tuning of the VSC and HPhP dispersion. Here, we report that the PMMA thickness defines the number of vibrational modes (N, Equation 1) available for coupling with the HPhP evanescent fields, whereas the hBN flake thickness affects the penetration depth of these fields and the interaction strength of the evanescent mode volume with the PMMA layer [43]. Further, we varied the isotopic composition of the boron in hBN to tune the frequency of the Reststrahlen bands relative to the PMMA vibrational frequencies and thus optimize the coupling strength within the system (Supporting Information S1: Figure S3).

## 2 Results and Discussion

The cavity designs employed in these experiments focused on decreasing V_m_, with four samples fabricated, all of which were comprised of a silicon substrate and a subsequent highly reflective 10 nm gold (Au) film. A thin film of PMMA was deposited onto the Au surface by spin coating, and an hBN flake was exfoliated onto the PMMA layer. The gold layer was utilized as a technique for electromagnetic confinement in the PMMA layer between gold and hBN. Per COMSOL simulations, theoretically, the confinement could induce coupling strengths up to 3 times that of the gold‐free model system (Figure 1C and Supporting Information S1: Figures S5–S6). Each sample contained multiple flake thicknesses due to the natural variance associated with the exfoliation method, thereby enabling the investigation of both the PMMA and hBN thicknesses within the same reduced sample set. Two samples were prepared with a 72‐nm PMMA layer but with varied boron isotope concentrations: 98.7% h^10^BN and 99.2% h^11^BN [37]. Additionally, two samples with 55 and 125 nm PMMA thicknesses were fabricated with h^10^BN. Note that using the reflective coating on the substrate and adjusting the thickness of the hBN flake layer both affect the magnitude of the evanescent mode volume, which directly affects the number of available vibrational modes present for coupling. The density of the polymer ($480\;MMA\;monomers/{nm}^{3}$) is assumed not to fluctuate throughout the organic layer, so the polymer thickness dictates the number of molecules present in the optical mode. A larger flake thickness results in a longer HPhP wavelength, an increased group velocity, and stronger coupling because of the increased evanescent mode extent.

Using hBN cavities (Figure 3A–F), mode volumes can be reduced to the order of tens to hundreds of nm^3^ in contrast to the previously mentioned FP cavity mode volumes that are on the order of several thousand μm^3^. The thickness of the hBN flake affects the polariton wavelength, which, within the 55‐nm h^11^BN flake (Figure 3D–F), exhibits a significantly larger compression factor than that found in the 110‐nm h^11^BN flake (Figure 3A–C). However, the slope of the dispersion plot (and thus the group velocity) also decreases with reduced hBN thickness. In the case of the 111‐nm h^11^BN flake, we observe a clear back bending in the experimental dispersion relation about the 1435‐cm^−1^ vibrational mode of PMMA (Figure 4A). The back bending is specific to the experimental data points and is seen when the relative *k* values begin to deviate from the data trend and “bend backward” on themselves to create the unique shape, as seen clearly in Figure 4A,C,D. This back bending is characteristic of VSC between the polaritonic mode and the CH_2_ bending mode of PMMA and corresponds to a pronounced Rabi splitting, indicative of strong coupling in the associated dispersion plot, seen in all panels of Figure 4 as the split in the trend of simulated data. In contrast, both the slope and the back bending of the dispersion curve are reduced for a 78‐nm h^11^BN system (Figure 4B), indicating that the reduced group velocity for the thinner flake results in a roughly 43% weaker coupling of modes. If the polymer thickness is varied instead of the flake thickness (for the purpose of qualitative comparison, flake thicknesses of 77 and 65 nm are considered adequately comparable here), increased back bending character is observed for the thicker PMMA layer; in the given example, Figure 4C exhibits coupling approximately 51% stronger than that observed in Figure 4D. We attribute this difference to an increase in the number of PMMA vibrational modes (N) available for VSC within the evanescent mode volume, another factor that increases the coupling strength (Equation 1).

**FIGURE 3:**
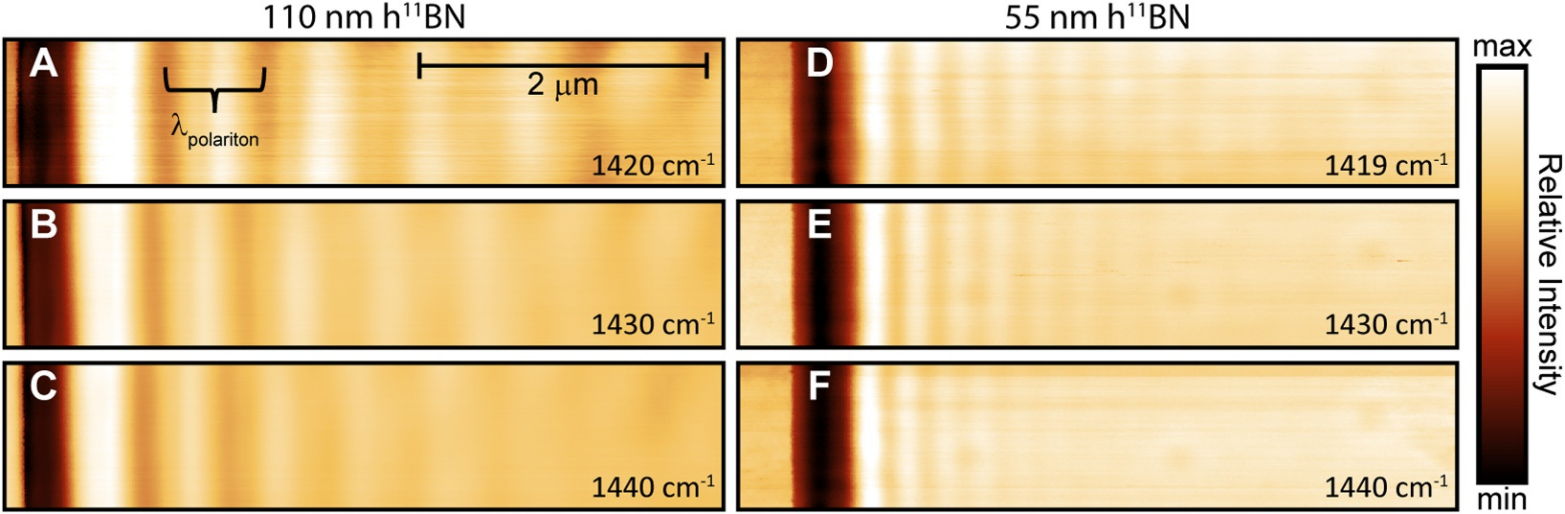
(A–C) s‐SNOM images of a 110‐nm h^11^BN flake on a 72‐nm layer of PMMA differing by 10 cm^−1^ in frequency with corresponding $\lambda_{\text{polariton}}$ of (A) 720 nm, (B) 650 nm, and (C) 601 nm. (D–F) s‐SNOM images of a 55‐nm h^11^BN flake on a 72‐nm layer of PMMA with corresponding $\lambda_{\text{polariton}}$ of (D) 344 nm, (E) 294 nm, and (F) 264 nm at free‐space frequencies comparable to those exhibited in Panels (A–C).

**FIGURE 4:**
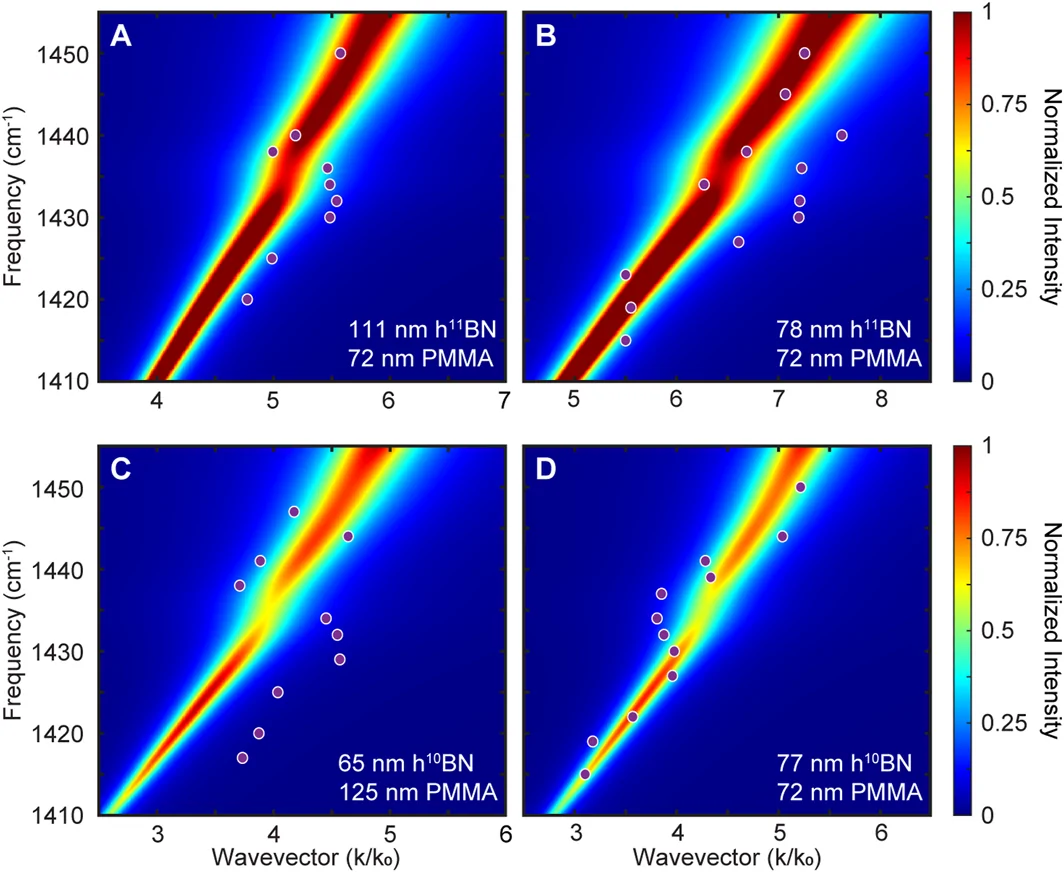
Simulated dispersion plots with experimental data points to examine the effects of cavity design on coupling to the 1435‐cm^−1^ mode of PMMA. PMMA thickness, hBN flake thickness, and hBN isotope compositions are varied as follows: (A) 72 nm PMMA and 111 nm h^11^BN, (B) 72 nm PMMA and 78 nm h^11^BN, (C) 125 nm PMMA and 65 nm h^10^BN, and (D) 72 nm PMMA and 77 nm h^10^BN.

The group velocity of these plots (Figures 4 and 5) is proportional to the slopes of the dispersion curves. These values were taken locally, as they do change with frequency. The calculated slope values of each of the dispersion plots were determined by first backing out the individual *k* values at set points in units of cm^−1^ so that a unitless slope could be obtained. This slope was then used in conjunction with the extracted Fourier transform values (Supporting Information S1: Figure S2) of the individual s‐SNOM image data points to determine polariton lifetimes (Table 1). Several trends emerge here. Most notably, a decreased slope yields a decreased polariton lifetime. Although we see a pronounced stronger coupling associated with the h^10^BN systems and attribute this to proximity to the TO phonon, the trends indicate that there is a range of lifetimes associated with the larger Rabi splittings reported; however, when zooming in to look specifically the systems containing h^10^BN and PMMA thicknesses less than 100 nm, we see a clear trend in the dataset that indicates an increased Rabi splitting associated with an increased group velocity, analogously, an increased slope value (Supporting Information S1: Figure S4E).

**FIGURE 5:**
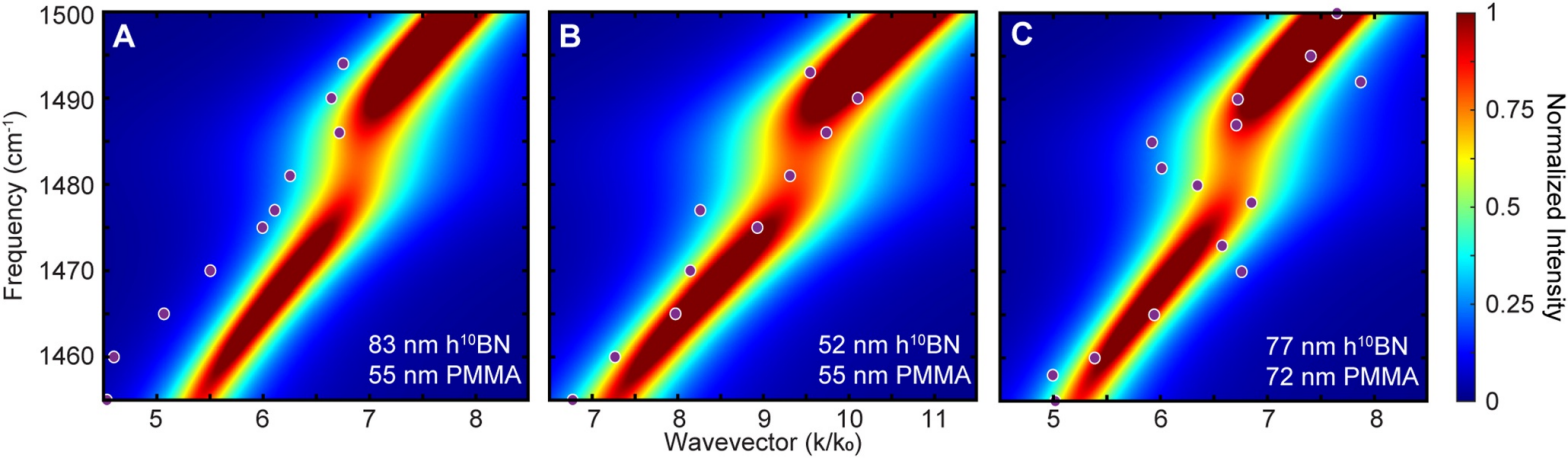
Simulated dispersion plots with experimental data points to examine the effects of cavity design on coupling to the 1483‐cm^−1^ mode of PMMA. PMMA thickness and h^10^BN flake thickness are varied as follows: (A) 55 nm PMMA and 83 nm h^10^BN, (B) 55 nm PMMA and 52 nm h^10^BN, and (C) 72 nm PMMA and 77 nm h^10^BN.

**TABLE 1:** Rabi splitting, polariton lifetime, and local slope values for reported polariton dispersions.

Dispersion plot	Local dω/dk		Polariton lifetime, *τ*		Rabi splitting	
	Upper branch	Lower branch	Upper branch (fs)	Lower branch (fs)	Δω (cm^−1^)	Δω (meV)
Figure 4A	0.10077	0.08597	251	294	8.0	0.988
Figure 4B	0.07109	0.06063	356	417	4.6	0.568
Figure 4C	0.07873	0.07708	321	328	9.4	1.160
Figure 4D	0.07656	0.06564	331	386	14.2	1.753
Figure 5A	0.05601	0.06239	452	406	13.4	1.654
Figure 5B	0.05181	0.04241	488	597	12.4	1.531
Figure 5C	0.05525	0.06164	458	411	13.6	1.679

The Reststrahlen band of hBN shifts to a lower frequency with increased isotopic mass, increasing the energy gap between the PMMA vibrational mode and the TO phonon (Figure 2). The PMMA mode thus interacts with a region of the TO dispersion curve at a larger wavevector, resulting in reduced coupling due to the corresponding lower group velocity. The impact of this shift is shown in Figure 4B,D. For otherwise comparable systems with equivalent flake and polymer thicknesses, the h^10^BN sample exhibits an approximate 78% increase in Rabi splitting within the dispersion compared to the h^11^BN sample.

The CH_3_ bending mode of PMMA appears at 1483 cm^−1^ and thus also falls within the Reststrahlen band of hBN. Just as with the 1435‐cm^−1^ mode, decreasing the thickness of the h^10^BN flake reduces the observed back bending character of the experimental data in the dispersion curve (Figure 5A,B), indicating an 8% weaker coupling, whereas increasing the thickness of the PMMA layer from 55 to 72 nm increases back bending character (Figure 5A,C) and thus coupling strength. The CH_3_ bending mode has a lower transition dipole strength (μ) than the CH_2_ bend at 1435 cm^−1^, which inherently reduces the coupling strength (Equation 1 and Supporting Information S1: Figure S4). Transition dipole strength calculation details can be found in the supplementary information. This reduction in coupling strength can be observed when comparing the back bending character and the distance between branches in the dispersion plots of the same system at different vibrational resonances (Figures 4, 5).

Although both the h^10^BN and h^11^BN isotopes used in the work exhibit Type II hyperbolic Reststrahlen bands that fully encompass the targeted vibrational resonances of 1435 cm^−1^ and 1483 cm^−1^ of PMMA, there was a clear increase in coupling strength in comparable systems with a smaller spectral distance from the TO phonon. This occurs because we see a reduction in group velocity and thus an increase in the kko value (Supporting Information S1: Figure S3). This phenomenon is well demonstrated simply by looking at the wavevector range needed to display the Rabi splitting in the plots of Figure 4. Supporting the idea that a target frequency resonance being closer to the TO phonon of the Reststrahlen band can yield stronger coupling within the evanescent modes, h^11^BN did exhibit stronger coupling at the lower target PMMA resonance than at the higher target PMMA resonance in comparison to the coupling strengths associated with the comparable h^10^BN system. That said, both isotopes were clearly capable of VSC with the targeted PMMA vibrational modes within their respective Reststrahlen bands.

The dispersion plots generated in MATLAB for the data overlays found in the main text were analyzed to locate the points of the Rabi splitting bounds associated with each of the target vibrational bonds in the work. Visually, the bounds are marked by a decrease in the intensity of the color red, but quantitatively, the bounds are determined as the location along the dispersion where the intensity drops abruptly from a normalized intensity of approximately 1 to a normalized intensity value of 0.9 or less. Several values were taken at each bound to ensure that the values were accurate. The *y*‐axis of these plots is the incident frequency in wavenumbers (cm‐1), so the distance between the bounds was determined and then converted to meV using a conversion factor of 8.1cm−11meV for a more straightforward comparison to existing strong coupling work. Table 1 contains these determined values, reporting them alongside their corresponding polariton lifetime values and dispersion plot slopes, which were used in the lifetime determination calculations (Supporting Information S1: Figure S4).

## 3 Conclusion

In this work, we have shown that several parameters predictably affect VSC in these subdiffractional cavity systems, which can provide guidance moving forward in experiments to increase coupling strength and apply VSC for determining the potential for modification of chemical reaction rates, pathways, and vibrational lifetimes. Extension to subdiffractional cavities offers even larger impact in the context of quantifying VSC of weaker dipoles, such as the PMMA modes investigated here. Isotopic enrichment of a hyperbolic material shifts the entire Reststrahlen band, which can also be employed to directly influence the coupling strength of the target resonance due to the inherent effects on the group velocity of the polariton as it moves away from the TO phonon. In the systems using h^10^BN, we observed increased coupling strength compared to comparable systems with h^11^BN due to the TO phonon of the band being closer to the target resonant PMMA frequencies. This is due to the more closely matched wavevector of the polariton propagation to the free‐space wavevector associated with the higher group velocity. Throughout this work, we investigated significant increases in coupling strength, with one of our systems (Figure 4D) showing a coupling strength 3 times that of another (Figure 4B) using this concept. With alterations of PMMA thicknesses, we saw slight variations in coupling strength, but with variations of flake thickness, we saw significant variations in coupling strength in the systems. A larger flake thickness led to a longer polariton wavelength, an increased group velocity, and stronger coupling. With a controllable hyperbolic flake thickness as an experimental parameter, this lays the groundwork for more direct control in future endeavors in strong coupling using phonon polariton evanescent modes of hyperbolic materials and weak organic vibrational modes. The coupling manipulation gives the tools to further increase coupling strength in novel systems or even to fine‐tune coupling of specific vibrational modes within a system.

## 4 Methodology

### 4.1 Scattering‐Type Scanning Near‐Field Optical Microscopy (s‐SNOM)

Two separate s‐SNOM systems were used for experimentation. The first system captured data at the 1483‐cm^−1^ Rabi splitting, but the quantum cascade laser (QCL) was unable to function in the frequency range needed for the 1435‐cm^−1^ splitting. The second system, located at Auburn University in Siyuan Dai's laboratory, was utilized specifically for work in the region of the lower Rabi splitting. s‐SNOM uses an atomic force microscopy (AFM) tip of platinum iridium to both measure and induce phonon polariton propagation by confining mid‐infrared (MIR) light to the near field. We use a pseudoheterodyning scheme for detection with a mercury cadmium telluride (MCT) detector. The s‐SNOM is used specifically for its ability to resolve down to approximately 10 nm, which allows for the direct imaging of the deeply subdiffractional nature seen with confinement in hyperbolic materials such as hBN. The detection scheme allows for the almost complete removal of far‐field signal from our measurements, leaving well‐defined near‐field information [44, 45, 46].

Second‐order harmonics were used for data analysis. This is both the first and cleanest harmonic dataset of each scan that gives negligible far‐field information. Generally, the second harmonic (O2A channel) was visually compared to the third and fourth harmonics [45, 46]. Note that the second‐, third‐, and fourth‐harmonic channels contain the same near‐field data but are easier to extract for Fourier transform analysis in cleaner scans (typically from the O2A channel).

### 4.2 Dispersion Plot Simulations

All dispersion plots, seen in Figures 2, 4, and 5, were generated using a MATLAB transfer matrix code put together by Nicolai Passler. The functionality of the code is detailed in the work by Passler et al. that details a matrix formalism for light propagation through anisotropic materials, specifically regarding phonon polariton propagation in polar dielectric heterostructures [47]. Within the code, there is an array generator for specific materials within the heterostructures. Dielectric function data for both hBN isotopic compositions were pulled from Giles et al. [25]. Dielectric function data for PMMA were pulled from Zhang et al. [48].

### 4.3 Sample Fabrication

Sample systems used in this work were prepared in the Vanderbilt Institute of Nanoscale Science and Engineering (VINSE). Sample systems were built on silicon substrates, cleaned using isopropanol and acetone, and then dried under nitrogen. These were then coated with 3 nm of chromium acting as an adhesion layer and 10 nm of gold using an electron beam coating technique. The gold‐coated substrates were then spin‐coated with PMMA using a solution of 950K A2 PMMA dissolved in anisole. Samples with 55, 72, and 125 nm polymer layers were created [49] using spin speeds of 5000, 3000, and 1000 rpm, respectively. Hexagonal boron nitride flakes were then exfoliated onto the samples using a simple tape exfoliation method. h^10^BN and h^11^BN flakes were both sourced from Kansas State University. Detailed fabrication methods are located in the supplementary information.

## Author Contributions

**Courtney L. Ragle:** conceptualization, writing – original draft, formal analysis, investigation, methodology, data curation, visualization. **John E. Buchner:** writing – review and editing, data curation. **In‐Ho Lee:** writing – review and editing, data curation. **Youngji Kim:** writing – review and editing, data curation. **Byung‐Il Noh:** writing – review and editing, data curation. **Saurabh Dixit:** writing – review and editing, data curation. **Joseph Matson:** writing – review and editing, data curation. **Jiahan Li:** writing – review and editing, data curation. **Siyuan Dai:** writing – review and editing, data curation. **J. H. Edgar:** writing – review and editing, data curation. **Sang‐Hyun Oh:** writing – review and editing, data curation. **Lauren E. Buchanan:** conceptualization, funding acquisition, writing – review and editing, methodology, validation, visualization, project administration, supervision, resources, data curation. **Joshua D. Caldwell:** conceptualization, funding acquisition, writing – review and editing, methodology, validation, visualization, project administration, supervision, resources, data curation.

## Supporting information


Supporting Information S1

## Data Availability

The data that support the findings of this study are available in the supplementary material of this article.
