# Supplementary material for: Tuning Vibrational Coupling Strength Between Hyperbolic Media and Organic Molecules
Source: Nanophotonics. 2026 Mar 24;15(6):e70051. doi: 10.1002/nap2.70051 (PMC13642090; doi:10.1002/nap2.70051)
Supplement: Supplementary file 1 [file NAP2-15-e70051-s001.docx]

**Supplementary Information**

*Tuning Vibrational Coupling Strength between Hyperbolic Media and Organic Molecules*

Courtney L. Ragle^1,2,3^, John E. Buchner^1,2^, In-Ho Lee^4^, Youngji Kim^2^, Byung-Il Noh^5^, Saurabh Dixit^2^, Joseph Matson^1,2^, Jiahan Li^6^, Siyuan Dai^5^, J. H. Edgar^6^, Sang-Hyun Oh^7^, Lauren E. Buchanan^3^, Joshua D. Caldwell^1,2,8,*^

^1^ Interdisciplinary Materials Science Program, Vanderbilt University, Nashville, TN, United States

^2^ Department of Mechanical Engineering, Vanderbilt University, Nashville, TN, United States

^3^ Department of Chemistry, Vanderbilt University, Nashville, TN, United States

^4^ Center for Quantum Technology, Korea Institute of Science and Technology (KIST), Seoul, 02792, Republic of Korea

^5^ Department of Mechanical Engineering, Auburn University, Auburn, AL, United States

^6^ Tim Taylor Department of Chemical Engineering, Kansas State University, Manhattan, KS, 66506 United States

^7^ Department of Electrical and Computer Engineering, University of Minnesota, Minneapolis, MN, United States

^8^ Sensorium Technological Laboratories, Nashville, TN, United States

*Corresponding Author: josh.caldwell@vanderbilt.edu

***s-SNOM Data Analysis***

Figure S1: s-SNOM images at edge of flake in system utilizing a polymer layer of 72 nm and an h^11^BN flake of 55 nm, showing clear polariton propagation at the denoted incident wavelength of each tile. The 2 µm scale bar is applicable to all tiles; the $\lambda_{polariton}$ marked in A) is specific to the marked tile to elucidate the decreasing size of the polariton wavelength when moving from A) to L).

Detail on s-SNOM operations and usage can be found in the main text. Each s-SNOM image is generated via a rastering of an AFM tip to yield images in which phonon polariton wave propagation is evident when the conditions are adequate such as those of our experimental systems (Fig. S1). When collecting experimental data to verify the behavior found in polariton dispersion plots, it is essential to have clear wave propagation because the averaged intensities are then extracted as “line scans” (Fig. S2A). These line scans show the intensities as the wave moves away from the edge of the launching or reflecting feature in the system. In this case we are seeing tip-launched waves as they are reflected off the edge of the flake. When the system is strongly coupled, there is a resonant mode absorbing strongly at a set frequency. This is where the rabi splitting arises in the simulated system dispersion plots and where our characteristic strong back-bending behavior arises in the experimental data. s-SNOM data was analyzed using Gwyddion [1] to extract the line scan intensity averaged across the flake width and OriginLab [2] software for the remainder of the analysis. The line scan data was then Fourier transformed (FT), yielding a value in units of m^-1^ (Fig. S2B). It should be noted that while the figure presented here to show the FT on a data set yields one prominent peak, it is often that multiple modes are present in the data and correspond to higher order modes that can be seen easily within the dispersion plots at higher $\frac{k}{k_{o}}$ values.

Figure S2: Two crucial steps in extrapolating wavevector data from individual s-SNOM images, A) Extraction of a line scan pulled directly from a s-SNOM image using Gwyddion software and plotted. B) Fourier transform analysis performed using preset operations in OriginLab software on the data set plotted in A.

The FT values are used to extrapolate associated adjusted wavevector $\frac{k}{k_{o}}$values with Eq. S1 at each incident wavelength, adjusted using the incident wavelength to give a unitless value $\frac{k}{k_{o}}$ that can be plotted for comparison on the corresponding dispersion plots as seen in Fig. 4 and 5 in the main text.

$$\left( \frac{k}{k_{o}} \right)= \frac{2 \times\lambda_{polariton}}{\pi\times\lambda_{incident}}$$

*(S1)*

$\lambda_{polariton}$ is the FT extacted value from processing using the described methodology and $\lambda_{incident}$ is the incident wavelength on the sample system as focused from the s-SNOM tip. The equation listed above is specific to an edge-launched polariton [3]. This methodology was used to extract and analyze the data collected from all samples in the study. Alternative methods, including certain types of sinusoidal fitting, can be used to extrapolate the same information once line scans have been extracted from the s-SNOM images.

Figure S3: Polariton dispersion plots specific to A-D) h^10^BN isotope and E-H) h^11^BN isotope. Each dispersion plot is some variation of a 55 nm or 110 nm hyperbolic material layer and a 55 nm or 110 nm polymer layer and is labelled on the respective tile. This assortment of dispersion plots highlights the effects of hyperbolic layer thickness on group velocities as well as the organic layer thickness. When moving from 55 nm to 110 nm hyperbolic layer as shown from A and B to C and D, as well as E and F to G and H, we see a significant increase in slope values of the shown first order polariton dispersion corresponding to group velocity. The shift of note between A-D and E-H is the shift in TO phonon frequency shown as the lower frequency bounds of the dispersions.

***Dispersion Plot Creation***

Dispersion plot simulations are detailed in the main text. To determine accurate values of the systems used in experiments, flake thickness measurements were made using a Bruker Asyst AFM tool in the Vanderbilt Institute of Nanoscale Science and Engineering (VINSE). These values were relevant to TMM simulation result accuracy and penetration depth calculation accuracies. Flake heights were extracted using Gwyddion software [1]. Using 2 scans of each flake, ten heights were extracted from each over the same region of the flake and averaged to give accurate values for use in the simulation plots. PMMA thicknesses were determined by the recipe used when spinning the material on and the gold thickness was determined similarly, although the gold thickness is irrelevant in the simulations due to its high reflectivity of electromagnetic fields. We designate strong coupling within these dispersion plots where we see a splitting of the dispersion line that is attributed to the spectral location of a vibrational resonance in the accompanying organic polymer.

***Polariton Lifetime Determination***

Utilizing information extracted directly from s-SNOM images (Fig. S2) and determining group velocity of the dispersion plots corresponding to those points, polariton lifetimes (Table 1) are determined as follows. The dispersion plots generated in MATLAB provide a y-axis of frequency in units cm^-1^ and an x-axis of normalized wavevector in $\frac{k}{k_{o}}$. To determine a localized group velocity, individual points were pulled directly above and directly below target Rabi splittings to derive a linear fit in terms of unnormalized k values in units matching the frequencies. The slope values, $\frac{\Delta\omega}{\Delta k}$, (unitless) can be multiplied by the speed of light to obtain the local group velocity in units of $(\frac{m}{s})$. Using a Lorentzian peak fit on the FFT data extracted from the SNOM wave data (e. g. Fig S2B). The full width half maximum value of the Fourier transformed data can be used in conjunction with the group velocity information to determine the local polariton lifetime, $\tau$.

$$\tau= {(V_{g}Im[k])}^{-1}$$

*(S2)*

The resulting value is in units of s, which is then easily converted to a more standardized polariton lifetime unit of ps.

**D**

**E**

h^10^BN System

Figure S4: Data points are organized into upper and lower polariton branches and hBN isotope for trend identification. Polariton lifetimes tend to show a longer lifetime in the corresponding lower polariton branch, which was expected. A) polariton lifetime as compared to corresponding PMMA thickness (nm) in system, B) polariton lifetime as compared to corresponding hBN thickness (nm) in system, C) polariton lifetime as compared to rabi splitting of system. D) polariton lifetime as compared to corresponding local dω/dk values, indicative of the dispersion plot slope. E) Rabi splittings associated with the h^10^BN systems studied that contained a PMMA thickness of less than 100 nm as compared to the non-normalized extracted dispersion plot slope values.

***Transition Dipole Strength calculations***

FTIR spectra taken of a sample made using the same PMMA as the samples fabricated for the work in the main text. Transition dipole strengths (TDS) were calculated for the two molecular resonances under investigation here, as well as the CO stretch of PMMA strictly for comparison to the strongest TDS value within the polymer. These calculations provided values that indicate the coupling strength dependence on dipole strength as clearly denoted in Eq. 1 of the main text. The values calculated for the resonances located at 1435 cm^-1^ (lower resonance) and 1483 cm^-1^ (upper resonance) are much smaller than the CO stretch, but this was both expected and a driver of the work. The lower resonance was calculated to have a TDS value at 12.1% of the CO vibrational resonance and the upper resonance was calculated to have a TDS value at 5% of the CO stretch.

Figure S5 A) FTIR reflectance spectrum of a PMMA sample prepared on a silicon substrate coated in gold at 950K. Inverted reflectance spectra of the B) CH_2_ and CH_3_ stretching region and the C) CO stretching region, with Gaussian fits of the main peaks. Gaussian fits were used to calculate dipole strength values of PMMA resonances.

After performing using Fourier transform infrared (FTIR) spectroscopy on a sample created solely for the purpose of analyzing the PMMA used in the production of the samples analyzed, individual dipole strengths were analyzed for several of the polymer modes for comparison. The two modes that coupled with the two isotopic compositions of h-BN were calculated to make sense of their relationship to each other and the CO stretch of PMMA located at 1725 cm^-1^ was analyzed for an overall comparison of dipole strengths within the polymer as the CO stretch has the highest transition dipole strength within the material and has been used in prior studies as the targeted resonance [4]. In the case of the peaks located at 1435 cm^-1^ and 1483 cm^-1^, the peaks were only partially resolved, so the calculation was performed for the half of the peak not containing overlap from unresolved spectra and doubled to find a true value. The following equation was used with a custom Python script [5]:

$$D=9.184 \times{10}^{-3}\int\frac{\epsilon(v)}{v}dv$$

*(S3)*

The calculations were made using an integration of the individual peaks of interest [6]. Due to humidity interference during PMMA spectral analysis, the peaks at 1435 cm^-1^ and 1483 cm^-1^ were fit to a gaussian (Fig. S4B), then calculated to obtain a viable comparison between dipole strengths.

Table S1: Dipole strength values extracted via gaussian fitting

| Peak | $\omega$ (cm^-1^) | Fit Type | Peak Area | Peak Height | FWHM |
| --- | --- | --- | --- | --- | --- |
| CH_2_ | 1435 | Gaussian | 5.31373 | 0.164 | 25.1 |
| CH_3_ | 1483 | Gaussian | 2.1826 | 0.109 | 30.5 |
| CO | 1725 | Gaussian | 44.0 | 2.296 | 18.0 |

***Upper Reststrahlen Band Considerations***

hBN of all isotopic compositions have a Type II hyperbolicity designated to an upper reststrahlen band, as seen in the main text. To illuminate the full dielectric behavior of the upper Reststrahlen band, see figure S7. This is an addition to the Reststrahlen band information found in figure 2 of the main text.

Figure S6: A) Dielectric function of h^10^BN plotted revealing the TO and LO phonon frequencies of the material to be at 1394 cm^-1^ and 1638 cm^-1^, respectively. B) Dielectric function of h^11^BN plotted revealing the TO and LO phonon frequencies of the material to be at 1360 cm^-1^ and 1599 cm^-1^, respectively.

***Lower Reststrahlen Band Considerations***

hBN of all isotopic compositions have a Type I hyperbolicity designated to a lower reststrahlen band. In the dielectric function of PMMA (Fig. S5A), there is a resonance located close to the lower reststrahlen bands of both h^10^BN and h^11^BN isotopes (Fig. S5B-C). However, while close in proximity, there is no spectral overlap and so no expected coupling. Due to the lack of spectral overlap, no analysis was performed in this Reststrahlen region. Due to the expected shifts present in the Reststrahlen bands of h^11^B^15^N, it is likely that strong coupling as that seen in the main text would occur in the lower reststrahlen band of this singular isotope of hBN.

Figure S7: A) dielectric function of PMMA plotted over the frequency range encompassing the lower Reststrahlen bands of h^10^BN and h^11^BN as seen in B) and C) respectively as plotted polariton dispersions.

***Sample Fabrication***

Sample systems used in this work were prepared in the Vanderbilt Institute of Nanoscale Science and Engineering (VINSE). Sample systems were built on silicon substrates diced to 10mm x 10mm using a DISCO DAD3200 dicing tool in VINSE. The substrates were thoroughly cleaned using isopropanol and acetone, then dried under nitrogen These were then coated with 3 nm of chromium acting as an adhesion layer and 10 nm of gold using an electron beam coating technique in the Angstrom Amod multimodal deposition chamber in VINSE. The gold coated substrates were then spin-coated with PMMA thin films using the A2 PMMA spin curves published by Azadi and Lopez as a guide for polymer layer thickness targets using a solution of 950K, A2 PMMA dissolved in anisole. Samples with 55, 72, and 125 nm polymer layers were created [7] using spin speeds of 5000 rpm, 3000 rpm, and 1000 rpm, respectively. Hexagonal boron nitride flakes were then exfoliated onto the samples using Ultron Systems, Inc. silicone-free adhesive plastic film of different boron isotopic compositions using a simple tape exfoliation method. h^10^BN and h^11^BN flakes were both sourced from the lab of James Edgar at Kansas State University. Only isotopically purified boron compositions were used for this work and due to the lower damping factor found as compared to that in naturally abundant hBN [8]. The thicknesses of the flakes measured using SNOM were verified using a Bruker Dimension Icon atomic force microscopy (AFM) tool in VINSE Each flake portion was measured twice in the AFM, then multiple measurements were made in Gwyddion software [1] and averaged together to verify flake thicknesses in the SNOM measurement areas.

Figure S8: A) COMSOL simulations of a system with 100 nm h10BN on 125 nm layer of PMMA on a (left) silicon substrate and a (right) gold-coated silicon substrate. The area above is area and the area below is air. The differences illustrate the confinement of the electric field when using the gold coating as was used for the systems analyzed in the main text. B-C) Dispersion plot highlighting the splitting present at the 1483 cm^-1^ for the system when on an uncoated silicon substrate (B) as compared to a gold-coated silicon substrate (C).

***Substrate Considerations***

Previous work using similar systems employed an organic layer deposited directly on a silicon substrate [9], [10]. In our work, we used a gold-coated silicon substrate to confine the electromagnetic field within the organic layer. COMSOL simulations of the systems with and without the gold layer (Fig. S6A) elucidate the confinement with the gold layer as compared visually to the electromagnetic leakiness of the Si substrate. Coupling strengths in simulated comparable systems are seen to be three times larger in the system containing the gold layer for confinement (Fig. S6B-C).

Figure S9: Near-field distributions of phonon polaritons in the hB10N/PMMA/metal structure at

wavenumbers (a) 1525 cm-1, (b) 1483 cm-1, and (c) 1435 cm-1. The thicknesses of the hB10N

and PMMA layers are 52 nm and 55 nm, respectively. The near fields are excited by an electric

dipole positioned 5 nm above the hB10N surface. (d) Power dissipation contributions from the

individual layers. ν1 and ν2 indicate the PMMA vibrational absorption peaks at 1435 cm-1 and

1483 cm-1, respectively.

***Electric field power dissipation***

The simulations were performed using hexagonal boron nitride enriched in the B10 isotope (h^10^BN). The heterostructure consisted of a 52 nm h^10^BN slab, a 55 nm PMMA overlayer, and an underlying gold film. The near-field distributions were excited by an oscillating electric dipole, positioned 5 nm above the h^10^BN surface and oriented parallel to the interface.

The analysis focused on wavenumbers corresponding to the PMMA vibrational absorption modes at ν₁ = 1435 cm^-1^ and ν₂ = 1483 cm^-1^, as well as the mid-band phonon frequency at 1525 cm^-1^ for comparison. At 1525 cm^-1^, a strong phonon polariton is launched, with near-fields highly concentrated within and around the h^10^BN layer, as shown in Fig. S7A. In contrast, at the vibrational frequencies, the near-fields are more confined within the PMMA layer rather than in h^10^BN (Figs. S7B and D). This trend is also evident in the power dissipation analysis (Fig. S7D), where dissipation in h^10^BN reaches a maximum at ν₂. As the frequency approaches the transverse optical phonon resonance of h^10^BN, the extinction coefficient of h^10^BN increases sharply, resulting in reduced power dissipation in h^10^BN at ν₁.

References:

[1]	D. Nečas and P. Klapetek, “Gwyddion: an open-source software for SPM data analysis,” *Open Phys.*, vol. 10, no. 1, pp. 181–188, Feb. 2012, doi: 10.2478/s11534-011-0096-2.

[2]	“OriginLab - Origin and OriginPro - Data Analysis and Graphing Software.” Accessed: May 28, 2025. [Online]. Available: https://www.originlab.com/

[3]	M. He *et al.*, “Guided Polaritons along the Forbidden Direction in MoO3 with Geometrical Confinement,” *Nano Lett.*, vol. 23, no. 11, pp. 5035–5041, June 2023, doi: 10.1021/acs.nanolett.3c00892.

[4]	A. D. Dunkelberger, B. S. Simpkins, I. Vurgaftman, and J. C. Owrutsky, “Vibration-Cavity Polariton Chemistry and Dynamics,” *Annu. Rev. Phys. Chem.*, vol. 73, pp. 429–451, Apr. 2022, doi: 10.1146/annurev-physchem-082620-014627.

[5]	“Introduction to Computation and Programming Using Python,” MIT Press. Accessed: May 28, 2025. [Online]. Available: https://mitpress.mit.edu/9780262542364/introduction-to-computation-and-programming-using-python/

[6]	M. Grechko and M. T. Zanni, “Quantification of transition dipole strengths using 1D and 2D spectroscopy for the identification of molecular structures via exciton delocalization: Application to α-helices,” *J. Chem. Phys.*, vol. 137, no. 18, p. 184202, Nov. 2012, doi: 10.1063/1.4764861.

[7]	M. Azadi, “PMMA A2 and A4 Spin Curves”.

[8]	M. He *et al.*, “Phonon engineering of boron nitride via isotopic enrichment,” *J. Mater. Res.*, vol. 36, no. 21, pp. 4394–4403, Nov. 2021, doi: 10.1557/s43578-021-00426-9.

[9]	A. Bylinkin *et al.*, “Real-space observation of vibrational strong coupling between propagating phonon polaritons and organic molecules,” *Nat. Photonics*, vol. 15, no. 3, Art. no. 3, Mar. 2021, doi: 10.1038/s41566-020-00725-3.

[10]	I. Dolado *et al.*, “Remote near-field spectroscopy of vibrational strong coupling between organic molecules and phononic nanoresonators,” *Nat. Commun.*, vol. 13, no. 1, p. 6850, Nov. 2022, doi: 10.1038/s41467-022-34393-4.
